# What digital health technology types are used in mental health prevention and intervention? Review of systematic reviews for systematization of technologies

**DOI:** 10.1093/joccuh/uiad003

**Published:** 2023-11-09

**Authors:** Naomichi Tani, Hiroaki Fujihara, Kenji Ishii, Yoshiyuki Kamakura, Mafu Tsunemi, Chikae Yamaguchi, Hisashi Eguchi, Kotaro Imamura, Satoru Kanamori, Noriko Kojimahara, Takeshi Ebara

**Affiliations:** Department of Ergonomics, Institute of Industrial Ecological Sciences, University of Occupational and Environmental Health, Kitakyushu 807-8555, Japan; Department of Ergonomics, Institute of Industrial Ecological Sciences, University of Occupational and Environmental Health, Kitakyushu 807-8555, Japan; The Ohara Memorial Institute for Science of Labour, Tokyo 151-0051, Japan; Department of Information Systems, Faculty of Information Science and Technology, Osaka Institute of Technology, Osaka 573-0196, Japan; Department of Occupational and Environmental Health, Nagoya City University Graduate School of Medical Sciences/Medical School, Nagoya 467-8601, Japan; Department of Nursing, Faculty of Nursing, Kinjo Gakuin University, Aichi 463-8521, Japan; Department of Mental Health, Institute of Industrial Ecological Sciences, University of Occupational and Environmental Health,Kitakyushu 807-8555, Japan; Department of Digital Mental Health, Graduate School of Medicine, The University of Tokyo, Tokyo 113-0033, Japan; Graduate School of Public Health, Teikyo University, Tokyo 173-8605, Japan; Section of Epidemiology, Shizuoka Graduate University of Public Health, Shizuoka 420-0881, Japan; Department of Ergonomics, Institute of Industrial Ecological Sciences, University of Occupational and Environmental Health, Kitakyushu 807-8555, Japan

**Keywords:** mental health, systematic review, digital health technology, digital phenotype, digital mental health intervention

## Abstract

Digital health technology has been widely applied to mental health interventions worldwide. Using digital phenotyping to identify an individual’s mental health status has become particularly important. However, many technologies other than digital phenotyping are expected to become more prevalent in the future. The systematization of these technologies is necessary to accurately identify trends in mental health interventions. However, no consensus on the technical classification of digital health technologies for mental health interventions has emerged. Thus, we conducted a review of systematic review articles on the application of digital health technologies in mental health while attempting to systematize the technology using the Delphi method. To identify technologies used in digital phenotyping and other digital technologies, we included 4 systematic review articles that met the inclusion criteria, and an additional 8 review articles, using a snowballing approach, were incorporated into the comprehensive review. Based on the review results, experts from various disciplines participated in the Delphi process and agreed on the following 11 technical categories for mental health interventions: heart rate estimation, exercise or physical activity, sleep estimation, contactless heart rate/pulse wave estimation, voice and emotion analysis, self-care/cognitive behavioral therapy/mindfulness, dietary management, psychological safety, communication robots, avatar/metaverse devices, and brain wave devices. The categories we defined intentionally included technologies that are expected to become widely used in the future. Therefore, we believe these 11 categories are socially implementable and useful for mental health interventions.

## Teaser

**Key points:**

- There was no consensus on the technical categories for digital health technologies, including digital phenotype.
- Through our literature review, we proposed 11 technical categories that include not only digital phenotypes but also technologies that are expected to become more prevalent in the future. However, this classification is a temporary one and requires continued discussion.
- This study will contribute to studies and policy-making in the area of digital mental health or e-Health.

## 1. A new occupational health strategy incorporating digital health technology is needed

The coronavirus disease pandemic led to widespread teleworking worldwide; however, it impaired the physical and mental health of workers.^1^ The World Health Organization (WHO) and International Labour Organization have called for occupational health services to provide ergonomic, mental, and psychosocial support using digital health technologies.^1^ Therefore, a new occupational health strategy incorporating digital health technologies is required in the global workplace.

In recent years, digital health technologies have evolved rapidly. According to the WHO, digital health is an expansion of the e-Health concept that encompasses a wider range of digital technologies for health, including smart and connected devices, the Internet of Things, advanced computing, big data analytics, and artificial intelligence (AI), including machine learning, and robotics.^2^ Moreover, digital health interventions, such as healthy worker decision support, tracking of health status using digital devices, and health education and training content in digital form for health professionals have been recommended by the WHO.^3^ We expect to observe an increasing number of developing countries adopting digital health technology interventions in the future.

The ergonomic perspective of a systems approach is helpful to develop new occupational health strategies that incorporate digital health technologies.^4^^,^^5^ The ergonomic systems approach is a method for optimizing well-being and performance (such as a comprehensive approach to stakeholders, the organizational environment, and the physical environment),^4^^,^^5^ and from this perspective it is necessary to understand technological trends, optimize the benefits and risks of new strategies, and implement appropriate strategies in society to develop proactive mental health measures using digital health technologies.

Wienert et al^6^ defined digital public health intervention as “an intervention that addresses at least one essential public health function through digital means,” which includes preventive behavior change, self-management, active monitoring, treatment, and diagnosis. A review of these digital public intervention studies showed that logs recorded by various sensors on smartphones are used for health outcomes, with a particular focus on mental health outcomes.^7^ As the ability to utilize digital health technologies in the mental health domain is of global interest, familiarity with technologies that are currently widely used or will be developed in the future is important.

Digital phenotypes are expected to enhance mental health measures^8^ as various intervention studies are being conducted. A digital phenotype is defined as “moment-by-moment quantification of the individual-level human phenotype in situ using data from personal digital devices”.^8^^,^^9^ Avant-garde works exist globally that utilize physical activity levels, sleep quality, and social behaviors for mental health measures based primarily on personal logs obtained from smartphones. Digital phenotypes can be classified as active or passive data.^8^ Active data are personal experiences captured through user engagement such as ecological momentary assessments.^8^^,^^10^ Passive data are automatically captured by accelerometers, GPS, and light sensors on smartphones and other devices to log an individual’s experience without user engagement.^8^^,^^10^ Conversely, some of the services provided are a mixture of active and passive data^11^; therefore, accurate classification may be difficult.^12^ Additionally, the terminologies used in previous studies were mixed, causing heterogeneity.^13^ Furthermore, in addition to the technology used for digital phenotyping, several other digital health technologies, including human-supported digital interventions, such as cognitive behavioral therapy (CBT) by therapists and others via teleconferences, the internet, and telephone^14^^,^^15^ are used in addressing mental health problems. Technologies including chatbots, gamification, and extended reality (eg, virtual, augmented, and mixed reality) are expected to be utilized as mental health measures in the future.^16^

Hence, no consensus exists on the classification of technologies that can be used to improve mental health. Moreover, from the viewpoint of occupational health technology developers and service providers, classifying the technology element levels is necessary to understand the availability of technologies applicable to mental health interventions. Therefore, we defined the intervention of digital health technologies in mental health as “healthcare services aimed at primary prevention provided to the general workforce using information and communication technology and digital technology. Digital health technology includes services that use technical algorithms (such as, application software [apps], communication robots, wearable devices, information provision through non-contact sensing devices, self-monitoring, and real-time feedback), and services that do not use technical algorithms (such as, online counseling that solely relies on internet-based means).”^17^

As mentioned above, various digital health technology representations include digital phenotypes.^13^ In other words, scoping and surveying digital health technology using only digital phenotypes results in bias. We also incorporate services that do not use technical algorithms into our definition of digital health technology. Therefore, a review is required that incorporates non-digital phenotypic articles to clarify overall digital health technologies. We conducted a review of digital phenotyping and other technologies to integrate and systematize the technologies that are currently being used or will be used in the mental health field.

## 2. Systematic review and Delphi technique process

This study implemented a review to investigate digital health technology trends used in digital phenotyping. Our review of articles on digital phenotyping was conducted in conformity with the Preferred Reporting Items for Systematic Reviews and Meta-Analyses (PRISMA) statement,^18^ and 2 databases were searched. First, we filtered PubMed by Review and Systematic Review and searched for articles in the last 5 years using the following terms: “digital phenotyp*” AND “mental health”. Further, we searched Google Scholar for the following terms: digital phenotype OR digital phenotyping AND mental health. After the search, following the removal of duplicates, articles were screened by 2 researchers (N.T. and T.E.). We defined the criteria for the inclusion of articles as follows: systematic reviews that collected and analyzed data for digital phenotyping of mental health, articles with content relevant to the technical classification, full articles, and articles published in the English language. The following exclusion criteria were used in selecting articles for screening: articles that were not about the technology used for digital phenotyping, systematic reviews not associated with mental health interventions, gray literature, or review protocols. Articles were selected based on the above selection criteria.

However, the lack of a detailed technical classification system with a consensus on digital phenotypes may have led to a selection bias. To cover digital health technologies related to mental health interventions, we needed technical information on digital phenotypes and technical information without digital phenotypes. Therefore, we adopted the snowballing method,^19^ which includes 2 types: forward snowballing, which identifies new papers based on those that cite the paper under study; and backward snowballing, which identifies new papers using a list of references. The backward snowballing method was used to identify new articles for inclusion in the reference list. Although this snowballing procedure continued until no new papers were identified, it was conducted only once. Namely, we utilized a 1-level backward snowballing approach and included additional articles. In this review, we attempted to identify the technologies used in digital health. We used the Delphi technique^20^^,^^21^ to identify digital health technologies. The Delphi method involves surveying several people with expertise in an issue and repeating the responses until group opinions converge. To systematize the technologies resulting from these reviews, we held discussions with experts in psychology and occupational health (C.Y., H.E., K.I., S.K., and N.K.), physiologists (H.F.), information scientists (Y.K.), labor scientists (K.I.), nurses (M.T.), physical therapists (N.T.), and ergonomists (T.E.) using the Delphi technique.

## 3. Systematization of digital health technologies and proposal

**Figure 1:**
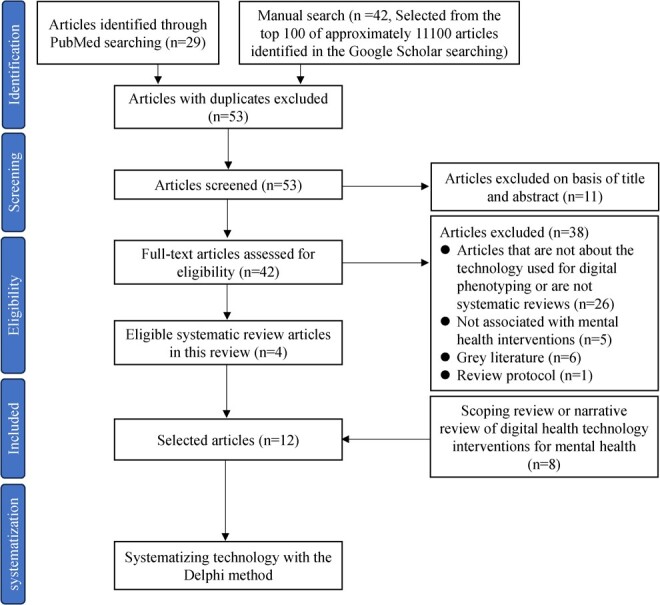
Flowchart of study selection.

The review process is illustrated in Figure 1. We extracted 71 articles from the 2 databases and removed 18 duplicates. The remaining 53 articles were screened after duplicates were removed. We extracted 42 articles based on their titles and abstracts. Furthermore, in a full-text review and applying the inclusion and exclusion criteria, we identified 4 articles that were systematic reviews of digital phenotyping for mental health interventions. In addition, we used a snowballing approach and added 8 review articles. Finally, we reviewed the digital health technologies for mental health interventions in 12 articles (Figure 1).

**Figure 2:**
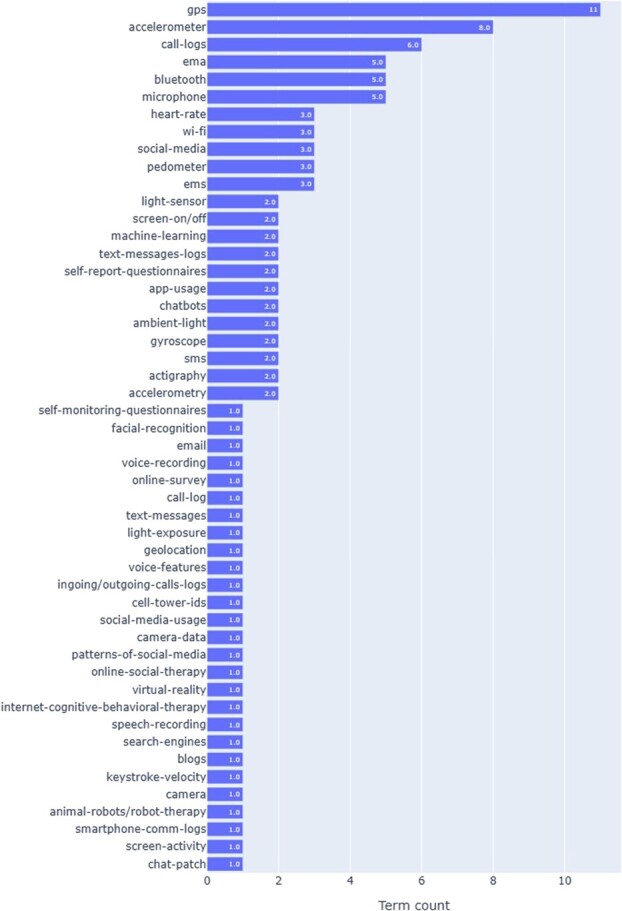
Visualization of technology terms extracted from the review. The technology term frequencies were counted using Natural Language Processing.

Table 1 summarizes the results of this review. Many studies mention technologies such as accelerometry,^10^^,^^11^^,^^13^^,^^22-28^ actigraphy,^26^^,^^29^ gyroscopy,^23^^,^^25^ pedometry,^23-25^^,^^27^ skin temperature,^24^^,^^25^ GPS,^10-13^^,^^22-29^ Wi-Fi,^11^^,^^13^^,^^23^^,^^25^ Bluetooth,^10-12^^,^^23^^,^^25^^,^^29^ microphone,^10^^,^^11^^,^^23^^,^^25^^,^^28^ heart rate sensor,^11^^,^^24^^,^^25^^,^^29^ light sensor,^10^^,^^11^^,^^13^^,^^23^^,^^25^^,^^26^ screen activity,^10-13^^,^^22^^,^^23^^,^^25^ call logs,^10-12^^,^^22^^,^^23^^,^^25-27^ email/text/SMS logs,^10-12^^,^^22-27^ app usage,^10^^,^^22^^,^^23^^,^^25^ social media logs,^12^^,^^22^^,^^24^^,^^25^^,^^27-29^ keystroke logs,^13^^,^^22^^,^^29^ self-report questionnaires,^12^^,^^22^^,^^24^^,^^26^ ecological momentary assessments/experience sampling methodology,^13^^,^^22^^,^^26-29^ facial recognition,^10^^,^^13^^,^^28^^,^^29^ voice recognition,^13^^,^^24^^,^^26^^,^^29^ chatbots,^24^^,^^28^^,^^29^ online therapy (such as CBT),^13^^,^^28^ robot therapy,^29^ and virtual reality.^28^ These technologies and techniques are used to identify, monitor, and improve an individual’s mood, physical activity, movement, communication, sleep status, and social activity for mental health. These technologies include a mixture of traditional and innovative technologies that at first glance are difficult to systematize. We also visualized technology terms using Natural Language Processing by Python (ver. 3.9.1) and found a lack of uniformity in referring to the same technology (Figure 2). However, we believe that a systematization approach based on the perspective of what specific mental health interventions are used when they are implemented is needed, that is, situations where the technologies are used.

**Table 1:** Summary of reviewed studies and technologies used for digital health technologies.

**No.**	**Ref.**	**First author**	**Year**	**Type of article**	**Technology and technique topics related to mental health interventions presented in the article**
1	11	Benoit J	2020	Systematic review	Accelerometer, GPS, microphone, heart rate sensor, ambient light sensor, touch screen touches, call logs, text/SMS logs, screen on/off, cell tower, Wi-Fi use, Bluetooth, screen unlock, charging status, and machine learning
2	12	Birk RH	2022	Review	Bluetooth, GPS, text message logs, call logs, screen activity logs, social media posts and timing of posts, and self-report questionnaires
3	22	Kamath J	2022	Review	EMA, self-report questionnaires, accelerometer, GPS, call logs, SMS text logs, screen active logs, social media logs, app logs, and computer-keyboard interactions
4	10	Melcher J	2020	Clinical review	Battery, camera events, browser history, Bluetooth, app usage, light sensor, lock/unlock, microphone, screen logs, SMS/email logs, call logs, accelerometer, and location
5	23	Mendes JPM	2022	Systematic review	Accelerometer, ambient light, app usage logs, battery level, Bluetooth encounters, call logs, cell tower ID, gyroscope, GPS, microphone, screen on/off, SMS text messages, step count, and Wi-Fi
6	24	Mouchabac S	2021	Review	Phone use, GPS, social media, temperature, heart rate, voice, sleep tracker, pedometer, accelerometer, questionnaire scales, SMS, and chatbot
7	25	Moura I	2023	Systematic review	Accelerometer, GPS, call logs, microphone, screen state, light sensor, SMS, Wi-Fi, gyroscope, app usage, heart rate, magnetometer, Bluetooth, social media, pedometer, and skin temperature
8	26	Saccaro LF	2021	Systematic review	SMS text, email, EMA, self-monitoring questionnaires, call log, text messages, actigraphy, light exposure, GPS, machine learning, accelerometer, geolocation, and voice features
9	27	Schick A	2023	Scoping review	EMS, pedometer, accelerometer, ingoing/outgoing call logs, text message logs, GPS, cell tower IDs, and social media usage
10	28	Torous J	2021	Review	EMA, accelerometry, GPS, microphone, camera data, patterns of social media, online social therapy, chatbots, virtual reality, and internet cognitive behavioral therapy
11	29	Van Assche E	2022	Review	EMA, EMS, speech recording, social media, search engines, blogs, Bluetooth, keystroke velocity, camera, actigraphy, GPS, heart rate, animal robots/robot therapy, and chatbots
12	13	Zarate D	2022	Systematic review	EMA, EMS, GPS, accelerometry, smartphone comm logs, screen activity, online survey, ambient light, voice recording, Wi-Fi, chat patch, and facial recognition

Abbreviations: EMA, ecological momentary assessments; ESM, experience sampling methodology; GPS, global positioning system; Ref., number in the References; SMS, short message service.

**Figure 3:**
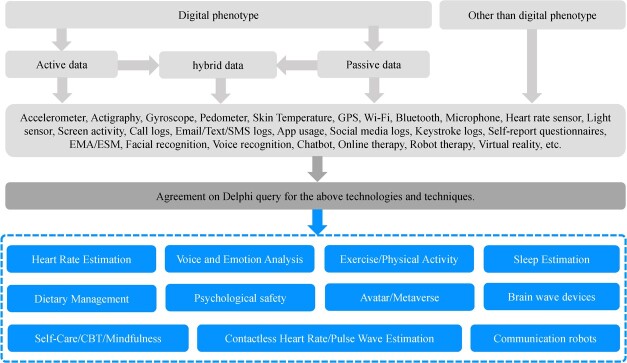
Systematization of technology using the Delphi method.

**Figure 4:**
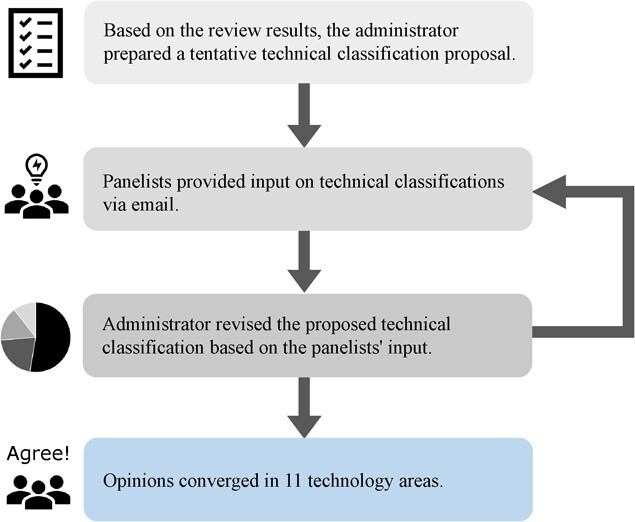
The Delphi process used in this study.

Figure 3 shows the 11 categories of technology areas that exist or are expected to become popular, as derived from the Delphi method. The Delphi process was conducted as follows: N.T. was the administrator, and other experts were assigned as panelists; based on the review results, the administrator prepared a tentative technical classification proposal, the panelists provided input on technical classifications via email, and the administrator revised the proposed technical classification based on the panelists’ input. The process was repeated until a convergence occurred of the opinion in the technology area on the issue “What are the digital health technology intervention situations and categories used to measure or monitor mental health?” (Figure 4). Using the Delphi method, the 11 categories we defined were heart rate estimation, exercise/physical activity, sleep estimation, contactless heart rate/pulse wave estimation, voice and emotion analysis, self-care/CBT/mindfulness, dietary management, psychological safety, communication robots, avatar/metaverse, and brain wave devices.

In this category, heart rate estimation, exercise/physical activity, and sleep estimation have reached a consensus in many reviews and are already in practical use.^10^^,^^11^^,^^13^^,^^22-29^ In a scoping review of health-related outcomes and digital phenotypes, 55% of articles utilized digital phenotyping for mental health interventions, whereas digital health technologies heavily used physical activity and sleep approaches.^7^ A large cohort study conducted in Norway reported that those who exercised at least 1 hour per week had a 12% lower incidence of future depression.^30^ Previous studies reported that people with versus without insomnia have approximately twice the risk of developing depression.^31^ This finding suggests that daily exercise and sleep monitoring are necessary for preventing mental health issues. Notably, the effectiveness of non-contact methods for measuring the heart rate has been reported in recent years.^32^ Thus, it seems inevitable that heart rate measurement would independently include contactless heart rate/pulse wave estimation, as well as contact measurement by wearable devices. An important aspect of mental health interventions is the use of microphones and cameras in smartphones to infer emotions through voice-based facial recognition.^10^^,^^11^^,^^13^^,^^23-26^^,^^28^^,^^29^ Voice and emotion analyses were included in this category because they are expected to be used frequently in the future for mood estimation.

Moreover, self-care/CBT/mindfulness and dietary management are expected to be internet-based^13^^,^^28^ and app-based^10^^,^^22^^,^^23^^,^^25^ mental health interventions. CBT and mindfulness can be conducted by a chatbot in an app or by a therapist online.^15^^,^^28^^,^^33^^,^^34^ Additionally, as digital health technology interventions are effective for eating disorders,^35^ online and app-based dietary approaches may become more extensively used in the future. Furthermore, organizational efforts are important for mental health,^36^ and interventions such as psychological safety (eg, crew resource management^37^) may be offered via apps in the future.

**Figure 5:**
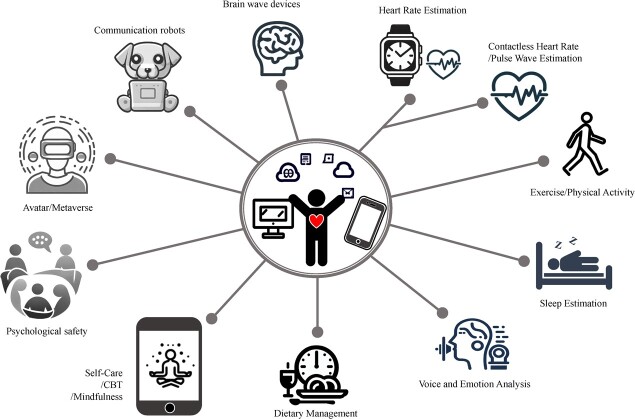
The 11 technology areas considered useful for mental health interventions.

In recent years, AI-powered chat bots have made headlines, indicating their potential for use in mental health interventions as well.^24^^,^^28^^,^^29^ As AI is increasingly used, mental health interventions for communication robots, such as animal robots and robot therapy, should also be considered.^29^ Presently, avatar therapy has not shown clear efficacy^38^ but may become a useful intervention as AI innovations continue to advance. Although no clear effect has been shown for extended reality,^16^^,^^34^ we have added the category of avatar or metaverse for the possibility that this may be an approach that holds promise for the future. Additionally, although not yet widely used, brain wave devices cannot be ignored in light of future technological innovations.^16^ Thus, the categories we defined intentionally included technologies that are expected to become widely used in the future. Therefore, we believe these 11 categories are socially implementable and useful for mental health interventions (Figure 5).

## 4. Conclusion

In this study, we tentatively systematized the categories in which digital health technologies contribute to mental health interventions. We believe that, from an occupational health perspective, the use of these technologies should not be limited to individual workers; rather, it should be employer-initiated as part of an organizational intervention. Meanwhile, it is apparent that the type of technology used by individuals, mainly digital phenotypes (eg, active and passive data), is currently widespread; however, the development of employer-friendly tools is lagging. In the field of occupational health, we must encourage developers to develop tools that employers can use to provide organizational interventions.

We reviewed articles that we deemed appropriate for classification as digital health technologies, including those that focused solely on technology, primary prevention, and treatment and those that are expected to become more widespread. This strategy was beneficial for covering various digital health technologies that had begun to spread rapidly. However, this is not limited to primary prevention articles that describe worker-only interventions. Innovation in digital health technology will continue to evolve faster than expected. As digital health technologies become more prevalent, we expect to see more articles focusing on primary prevention among workers in the occupational health field. Therefore, the systematization made in this study is only a temporary classification to organize the current situation, and the 11 categories should be updated periodically. In the future, it will be necessary to further elaborate on systematization by discussing it with stakeholders surrounding digital health technologies and utilizing the Patient and Public Involvement framework.

## Author contributions

N.T. and T.E. contributed to the design of this study. N.T. performed the data collection. N.T. drafted the manuscript. All authors discussed data classification. T.E. supervised the study. All authors contributed to the writing of the manuscript, critical revision, and agreed with the final version.

## Funding

This work was supported by the Japan Agency for Medical Research and Development (AMED) under Grant Number JP22rea522006.

## Conflicts of interest

K.I. is employed at the Department of Digital Mental Health, an endowment department supported by an unrestricted grant from 15 enterprises (https://dmh.m.u-tokyo.ac.jp/c), outside the submitted work. T.E. has received research support from ES Japan Inc., developing a solution business using voice-emotional signature analysis. There are no conflicts of interest to report for the other authors.

## Data availability

The data underlying this article will be shared on reasonable request to the corresponding author.

## References

[1] World Health Organization and International Labour Organization. *Healthy and Safe Telework*. Geneva: World Health Organization and the International Labour Organization; February 2022. Accessed July 9, 2023. https://www.ilo.org/global/publications/books/WCMS_836250/lang--en/index.htm

[2] World Health Organization . Global Strategy on Digital Health 2020–2025. August 2021. Accessed June 29, 2023. https://apps.who.int/iris/handle/10665/344249

[3] World Health Organization . Recommendations on Digital Interventions for Health System Strengthening. June 2019. Accessed June 29, 2023. https://www.who.int/publications/i/item/9789241550505 31162915

[4] International Ergonomics Association . Core Competencies in Human Factors and Ergonomics. August 2021. Accessed July 10, 2023. https://iea.cc/revision-of-core-competencies-in-human-factors-and-ergonomics/

[5] Ebara T , Toriizuka T, Kotani K, Fujita Y. Three things HFE professionals should practice: learning from the revised Core Competencies in Human Factors and Ergonomics by International Ergonomics Association. Japan J Ergonomics. 2021;57(4):155-164. 10.5100/jje.57.155

[6] Wienert J , Jahnel T, Maaß L. What are digital public health interventions? First steps toward a definition and an intervention classification framework. J Med Internet Res. 2022;24(6):e31921. 10.2196/31921 35763320 PMC9277526

[7] Lee K , Lee TC, Yefimova M, et al. Using digital phenotyping to understand health-related outcomes: a scoping review. Int J Med Inform. 2023;174:105061. 10.1016/j.ijmedinf.2023.105061 37030145

[8] Onnela JP , Rauch SL. Harnessing smartphone-based digital phenotyping to enhance behavioral and mental health. Neuropsychopharmacology. 2016;41(7):1691-1696. 10.1038/npp.2016.7 26818126 PMC4869063

[9] Torous J , Kiang MV, Lorme J, Onnela JP. New tools for new research in psychiatry: a scalable and customizable platform to empower data driven smartphone research. JMIR Ment Health. 2016;3(2):e16. 10.2196/mental.5165 27150677 PMC4873624

[10] Melcher J , Hays R, Torous J. Digital phenotyping for mental health of college students: a clinical review. Evid Based Ment Health. 2020;23(4):161-166. 10.1136/ebmental-2020-300180 32998937 PMC10231503

[11] Benoit J , Onyeaka H, Keshavan M, Torous J. Systematic review of digital phenotyping and machine learning in psychosis spectrum illnesses. Harv Rev Psychiatry. 2020;28(5):296-304. 10.1097/HRP.0000000000000268 32796192

[12] Birk RH , Samuel G. Digital phenotyping for mental health: reviewing the challenges of using data to monitor and predict mental health problems. Curr Psychiatry Rep. 2022;24(10):523-528. 10.1007/s11920-022-01358-9 36001220

[13] Zarate D , Stavropoulos V, Ball M, de Sena CG, Jacobson NC. Exploring the digital footprint of depression: a PRISMA systematic literature review of the empirical evidence. BMC Psychiatry. 2022;22(1):421. 10.1186/s12888-022-04013-y 35733121 PMC9214685

[14] Kane H , Gourret Baumgart J, El-Hage W, et al. Opportunities and challenges for professionals in psychiatry and mental health care using digital technologies during the COVID-19 pandemic: systematic review. JMIR Hum Factors. 2022;9(1):e30359. 10.2196/30359 34736224 PMC8820762

[15] Tremain H , McEnery C, Fletcher K, Murray G. The therapeutic alliance in digital mental health interventions for serious mental illnesses: narrative review. JMIR Ment Health. 2020;7(8):e17204. 10.2196/17204 32763881 PMC7442952

[16] De Witte NAJ , Joris S, Van Assche E, Van Daele T. Technological and digital interventions for mental health and wellbeing: an overview of systematic reviews. Front Digit Health. 2021;3:754337. 10.3389/fdgth.2021.754337 35005695 PMC8732948

[17] Eguchi H , Kojimahara N, Kanamori S, et al. The use of digital health technology to provide mental health services for employees in Japan. Environ Occup Health Pract. 2023;e17204. Forthcoming. 10.1539/eohp.2023-0016-CT PMC11841777 40061366

[18] Moher D , Liberati A, Tetzlaff J, Altman DG, The PRISMA Group. Preferred reporting items for systematic reviews and meta-analyses: the PRISMA statement. PLoS Med. 2009;6(7):e1000097. 10.1371/journal.pmed.1000097 19621072 PMC2707599

[19] Greenhalgh T , Peacock R. Effectiveness and efficiency of search methods in systematic reviews of complex evidence: audit of primary sources. BMJ. 2005;331(7524):1064-1065. 10.1136/bmj.38636.593461.68 16230312 PMC1283190

[20] Hohmann E , Brand JC, Rossi MJ, Lubowitz JH. Expert opinion is necessary: Delphi panel methodology facilitates a scientific approach to consensus. Arthroscopy. 2018;34(2):349-351. 10.1016/j.arthro.2017.11.022 29413182

[21] Martinez-Martin N , Greely HT, Cho MK. Ethical development of digital phenotyping tools for mental health applications: Delphi study. JMIR Mhealth Uhealth. 2021;9(7):e27343. 10.2196/27343 34319252 PMC8367187

[22] Kamath J , Leon Barriera R, Jain N, Keisari E, Wang B. Digital phenotyping in depression diagnostics: integrating psychiatric and engineering perspectives. World J Psychiatry. 2022;12(3):393-409. 10.5498/wjp.v12.i3.393 35433319 PMC8968499

[23] Mendes JPM , Moura IR, Van de Ven P, et al. Sensing apps and public data sets for digital phenotyping of mental health: systematic review. J Med Internet Res. 2022;24(2):28735. 10.2196/28735 PMC8895287 35175202

[24] Mouchabac S , Conejero I, Lakhlifi C, et al. Improving clinical decision-making in psychiatry: implementation of digital phenotyping could mitigate the influence of patient's and practitioner's individual cognitive biases. Dialogues Clin Neurosci. 2021;23(1):52-61. 10.1080/19585969.2022.2042165 35860175 PMC9286737

[25] Moura I , Teles A, Viana D, Marques J, Coutinho L, Silva F. Digital phenotyping of mental health using multimodal sensing of multiple situations of interest: a systematic literature review. J Biomed Inform. 2023;138:104278. 10.1016/j.jbi.2022.104278 36586498

[26] Saccaro LF , Amatori G, Cappelli A, Mazziotti R, Dell'Osso L, Rutigliano G. Portable technologies for digital phenotyping of bipolar disorder: a systematic review. J Affect Disord. 2021;295:323-338. 10.1016/j.jad.2021.08.052 34488086

[27] Schick A , Rauschenberg C, Ader L, et al. Novel digital methods for gathering intensive time series data in mental health research: scoping review of a rapidly evolving field. Psychol Med. 2023;53(1):55-65. 10.1017/S0033291722003336 36377538 PMC9874995

[28] Torous J , Bucci S, Bell IH, et al. The growing field of digital psychiatry: current evidence and the future of apps, social media, chatbots, and virtual reality. World Psychiatry. 2021;20(3):318-335. 10.1002/wps.20883 34505369 PMC8429349

[29] Van Assche E , Antoni Ramos-Quiroga J, Pariante CM, et al. Digital tools for the assessment of pharmacological treatment for depressive disorder: state of the art. Eur Neuropsychopharmacol. 2022;60:100-116. 10.1016/j.euroneuro.2022.05.007 35671641

[30] Harvey SB , Øverland S, Hatch SL, Wessely S, Mykletun A, Hotopf M. Exercise and the prevention of depression: results of the HUNT cohort study. Am J Psychiatr. 2018;175(1):28-36. 10.1176/appi.ajp.2017.16111223 28969440

[31] Baglioni C , Battagliese G, Feige B, et al. Insomnia as a predictor of depression: a meta-analytic evaluation of longitudinal epidemiological studies. J Affect Disord. 2011;135(1-3):10-19. 10.1016/j.jad.2011.01.011 21300408

[32] Chan PY , Ryan NP, Chen D, McNeil J, Hopper I. Novel wearable and contactless heart rate, respiratory rate, and oxygen saturation monitoring devices: a systematic review and meta-analysis. Anaesthesia. 2022;77(11):1268-1280. 10.1111/anae.15834 35947876

[33] McIntyre RS , Greenleaf W, Bulaj G, et al. Digital health technologies and major depressive disorder. CNS Spectr. 2023. 1-12. 10.1017/S1092852923002225 37042341

[34] Balcombe L , De Leo D. Evaluation of the use of digital mental health platforms and interventions: scoping review. Int J Environ Res Public Health. 2023;20(1):362. 10.3390/ijerph20010362 PMC9819791 36612685

[35] Kim M , Choi HJ. Digital therapeutics for obesity and eating-related problems. Endocrinol Metab. 2021;36(2):220-228. 10.3803/EnM.2021.107 PMC8090472 33761233

[36] Tsutsumi A , Nagami M, Yoshikawa T, Kogi K, Kawakami N. Participatory intervention for workplace improvements on mental health and job performance among blue-collar workers: a cluster randomized controlled trial. J Occup Environ Med. 2009;51(5):554-563. 10.1097/JOM.0b013e3181a24d28 19365287

[37] Serou N , Sahota LM, Husband AK, Forrest SP, Slight RD, Slight SP. Learning from safety incidents in high-reliability organizations: a systematic review of learning tools that could be adapted and used in healthcare. Int J Qual Health Care. 2021;33(1):046. 10.1093/intqhc/mzab046 PMC8271183 33729493

[38] Aali G , Kariotis T, Shokraneh F. Avatar therapy for people with schizophrenia or related disorders. Cochrane Database Syst Rev. 2020; 5(5):CD011898. 32413166 10.1002/14651858.CD011898.pub2 PMC7387758

